# Regulation of the arachidonic acid mobilization in macrophages by combustion-derived particles

**DOI:** 10.1186/1743-8977-8-23

**Published:** 2011-08-02

**Authors:** Susanne Fritsch-Decker, Tanja Both, Sonja Mülhopt, Hanns-Rudolf Paur, Carsten Weiss, Silvia Diabaté

**Affiliations:** 1 Karlsruhe Institute of Technology, Campus North, Institute of Toxicology and Genetics, Hermann-von-Helmholtz-Platz 1, 76344 Eggenstein-Leopoldshafen, Germany; 2 Karlsruhe Institute of Technology, Campus North, Institute of Technical Chemistry, Hermann-von-Helmholtz-Platz 1, 76344 Eggenstein-Leopoldshafen, Germany

## Abstract

**Background:**

Acute exposure to elevated levels of environmental particulate matter (PM) is associated with increasing morbidity and mortality rates. These adverse health effects, e.g. culminating in respiratory and cardiovascular diseases, have been demonstrated by a multitude of epidemiological studies. However, the underlying mechanisms relevant for toxicity are not completely understood. Especially the role of particle-induced reactive oxygen species (ROS), oxidative stress and inflammatory responses is of particular interest.

In this *in vitro *study we examined the influence of particle-generated ROS on signalling pathways leading to activation of the arachidonic acid (AA) cascade. Incinerator fly ash particles (MAF02) were used as a model for real-life combustion-derived particulate matter. As macrophages, besides epithelial cells, are the major targets of particle actions in the lung murine RAW264.7 macrophages and primary human macrophages were investigated.

**Results:**

The interaction of fly ash particles with macrophages induced both the generation of ROS and as part of the cellular inflammatory responses a dose- and time-dependent increase of free AA, prostaglandin E_2_/thromboxane B_2 _(PGE_2_/TXB_2_), and 8-isoprostane, a non-enzymatically formed oxidation product of AA. Additionally, increased phosphorylation of the mitogen-activated protein kinases (MAPK) JNK1/2, p38 and ERK1/2 was observed, the latter of which was shown to be involved in MAF02-generated AA mobilization and phosphorylation of the cytosolic phospolipase A_2_. Using specific inhibitors for the different phospolipase A_2 _isoforms the MAF02-induced AA liberation was shown to be dependent on the cytosolic phospholipase A_2_, but not on the secretory and calcium-independent phospholipase A_2_. The initiation of the AA pathway due to MAF02 particle exposure was demonstrated to depend on the formation of ROS since the presence of the antioxidant N-acetyl-cysteine (NAC) prevented the MAF02-mediated enhancement of free AA, the subsequent conversion to PGE_2_/TXB_2 _via the induction of COX-2 and the ERK1/2 and JNK1/2 phosphorylation. Finally we showed that the particle-induced formation of ROS, liberation of AA and PGE_2_/TXB_2 _together with the phosphorylation of ERK1/2 and JNK1/2 proteins was decreased after pre-treatment of macrophages with the metal chelator deferoxamine mesylate (DFO).

**Conclusions:**

These results indicate that one of the primary mechanism initiating inflammatory processes by incinerator fly ash particles seems to be the metal-mediated generation of ROS, which triggers via the MAPK cascade the activation of AA signalling pathway.

## Background

Over the last decades a multitude of epidemiological studies could correlate elevated levels of environmental particulate matter (PM) with increasing cardiorespiratory morbidity and mortality rates [1,2], predominantly in susceptible individuals or humans with pre-existing pulmonary or cardiovascular diseases [3-6]. Inflammation is considered as a major factor contributing to adverse health effects in response to elevated concentrations of ambient PM and nanoparticles [7-10]. Furthermore, the respiratory and systemic inflammatory effects have been associated with the induction of oxidative stress [11,12].

Alveolar macrophages, besides epithelial cells, are the major targets of particle actions in the lung and play a key role in particle-induced inflammation and lung diseases. Thus, it has been shown *in vitro *that bronchial epithelial cells as well as alveolar macrophages release interleukin (IL)-8, and tumor necrosis factor-α (TNF-α) in response to respirable particles [13-16]. In addition, treatment of monocytes and macrophages with PM results in an increased liberation of arachidonic acid and enhances formation of inflammatory mediators [17-19].

Arachidonic acid (AA) released from membrane phospholipids by phospholipases A_2 _(PLA_2_) serves as the precursor for a family of lipid mediators formed by oxygenation through the cyclooxygenase (COX) and lipoxygenase (LOX) pathways. The generation of lipid mediators, also called eicosanoids, plays a central role in cellular homeostasis, host defense and inflammatory processes. Therefore, a deregulation of AA metabolism can lead to the development of many oxidative stress related diseases such as pulmonary fibrosis and lung cancer [20-23]. Oxidants such as H_2_O_2 _have been reported to trigger AA release and its metabolism, involving multiple enzymes and pathways [24-26]. In this context, various studies revealed, that particles trigger the generation of reactive oxygen species and oxidative stress, resulting in an increased production of inflammatory mediators [27,28]. Brown and colleagues [29] demonstrated in primary alveolar macrophages and human monocytes that exposure to ultrafine carbon black particles triggers nuclear translocation of the transcription factor NF-κB as well as an increased TNF-α protein release, two responses which were reduced by the antioxidant nacystelin (NAL). In addition, the antioxidant N-acetyl-cysteine (NAC) also suppressed the cyclooxygenase-2 (COX-2) induction, prostaglandin E_2 _(PGE_2_) synthesis and activation of the transcription factor NF-κB by organic components of combustion derived particles, emphasizing the important role of ROS in particle-mediated inflammation [30]. Several studies supported an influence of transition metals, which are abundant constituents of ambient particulate matter, in mediating particle-induced formation of ROS [31]. Voelkel *et al*. [32] demonstrated a protective effect of the metal chelator DFO on fly-ash-induced formation of ROS. Furthermore, human studies have shown that the instillation of extracts of PM with a high metal content induced a stronger influx of inflammatory cells compared with particles with smaller metal content [33].

Recently, Beck-Speier *et al*. [34] reported that extracellularly insoluble Fe_2_O_3 _particles are partly soluble intracellularly which modulates the particle-mediated IL-6 and PGE_2 _release *in vitro *and *in vivo*. This demonstrates that even small amounts of bioavailable metals are able to activate inflammatory processes including the arachidonic acid cascade.

In a previous study we used the fly ash MAF02 originating from a municipal waste incinerator facility as a model for real-life combustion-derived particulate matter (PM) to study the *in vitro *responses in RAW264.7 macrophages [17]. We have shown that MAF02 particles induced an increased mobilization of AA and enhanced expression of COX-2 protein. Furthermore, the fly ash-induced AA mobilization was shown to be dependent on activation of the mitogen-activated protein kinases (MAPKs) ERK1/2 and to a lesser extent on p38. These processes were accompanied by the intracellular formation of ROS which resulted in the upregulation of various oxidative stress markers such as the increase of the cellular glutathione (GSH) content and the induction of the antioxidative enzyme heme oxygenase-1 (HO-1). The main focus of the present study was to elucidate the role of ROS in the activation of AA cascade in macrophages, and to examine which constituents of MAF02 particles are responsible for the cellular effects.

Our results revealed that exposure to MAF02 particles induces an activation of the arachidonic acid cascade in the murine macrophage cell line RAW264.7 as well as in human primary monocyte-derived macrophages (MDM) which was correlated with particle uptake into the cells. Particle-induced mobilization of AA requires the activation of ERK1/2 and is mediated through the activation of the cytosolic phospholipase A_2 _(cPLA_2_). Furthermore, initiation of the AA cascade is dependent on the formation of ROS. Analysis of the signalling pathways demonstrated that pre-treatment of macrophages with the antioxidant NAC leads to a significantly reduced mobilization of AA accompanied by a decreased activation of the ERK1/2 and JNK1/2 pathways as well as reduced induction of COX-2 and release of inflammatory lipid mediators. In addition, the metal chelator DFO prevented the MAF02-induced generation of ROS, the activation of downstream MAPK signalling and the arachidonic acid cascade. In summary, these data provide evidence for the involvement of metal-derived ROS formation in mediating particle-induced initiation of the inflammatory arachidonic acid cascade.

## Results

### Particle characterization

**Figure 1:**
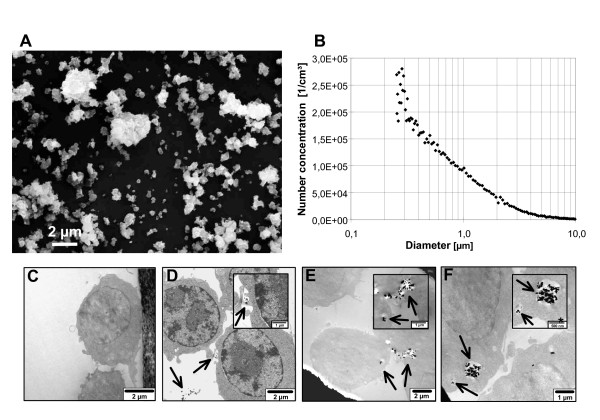
**Characterization and uptake of MAF02 particles**. The fly ash was resuspended in filtered air, deposited on a Nuclepore™ membrane and analyzed by SEM (A). The bar indicates 2 μm. The aerosol was additionally measured by PCS-2000 light scattering spectrometer (B). The graph is representative of three measurements. For TEM analysis RAW264.7 cells were left untreated (C) or exposed to 50 μg/ml MAF02 particles (10.4 μg/cm^2^) for 1 h (D), 2.5 h (E) or 5 h (F). Representative images of three different experiments are shown. Arrows indicate MAF02 particles in agglomerates of different sizes.

The MAF02 fly ash particles used in this study as a model for combustion-derived PM are composed of a large number of components. The water-soluble fraction of MAF02 was determined to 61% by weight when extracted with deionised water. Elemental analysis revealed that the major components of MAF02 were Na, K, and Ca which mainly occur as water-soluble salts (chlorides and sulphates). The major trace metals in MAF02 fly ash were Zn, Pb and Fe which amount to 15.2, 5.1 and 1.7% of weight, respectively. 63% of Zn, 81% of Pb and 87% of Fe were not extractable with water. Elemental carbon amounted to 7 mg/g. Previous analysis of the particle size distribution by scanning mobility particle sizing (SMPS) after resuspension in air showed that the number concentration was dominated by fine and ultrafine particles with a modal value of 165 nm [13]. Additional analyses using a light scattering spectrometer detecting the particle sizes up to 10 μm (PCS-2000) and scanning electronic microscopy (SEM) of deposited particles confirmed the low percentage of large agglomerates (Figure 1A-B).

### Uptake of fly ash particles

In order to examine the uptake and cellular distribution of MAF02 particles in macrophages, transmission electron microscopy (TEM) images of murine RAW264.7 cells, incubated with 50 μg/ml (10.4 μg/cm^2^) fly ash particles, were analyzed. TEM analysis demonstrated that MAF02 fly ash particles were taken up by RAW264.7 macrophages in dependence of time within the exposure period of 1 h up to 5 h (Figure 1C-F). The images present single and agglomerated particles in the intracellular space. Some are surrounded by a membrane, others not. No particles were observed within mitochondria or nuclei.

### Viability and ROS production in fly-ash treated RAW264.7 cells in comparison to human MDM

**Figure 2:**
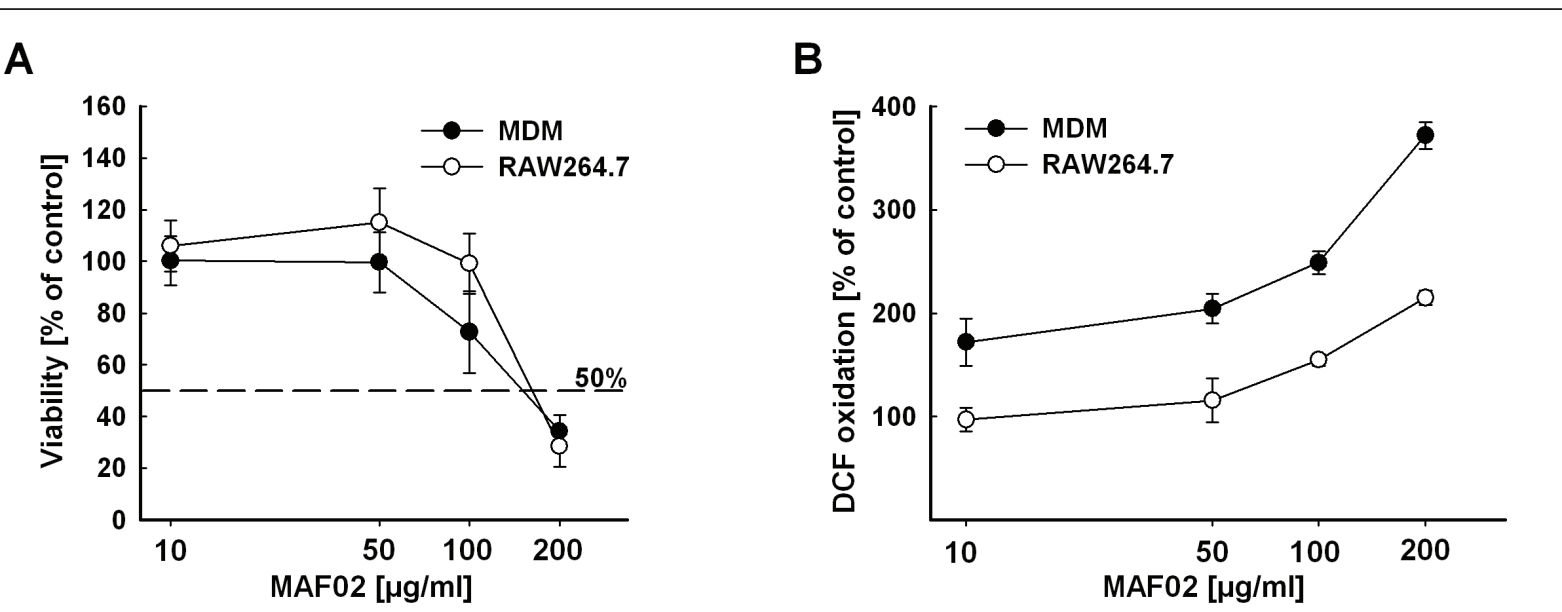
**MAF02 reduces the viability of human MDM and RAW264.7 macrophages and induces intracellular ROS in dependence of dose**. (A) For testing viability human MDM and RAW264.7 macrophages were treated with MAF02 particles at 10, 50, 100 and 200 μg/ml (6.3, 31.3, 62.5 and 125 μg/m^2^) for 20 hours. The viability was analyzed using the WST-1 assay and results are given in percentage of untreated controls. (B) For studying ROS formation macrophages were treated with the same particle concentrations for 3 h and subsequently loaded with H_2_DCF. Induction of ROS was detected by fluorescence measurement of DCF and changes in fluorescence intensity are expressed as percentage of untreated controls. Results are the mean ± s.e.m. of three independent experiments, each carried out in triplicate.

The dose-response curves of the effects of MAF02 particles on the viability and the intracellular ROS formation of RAW264.7 macrophages are published in Fritsch *et al*. [17]. To determine whether the transformed murine macrophage cell line represents an appropriate *in vitro *model system, we compared the dose-response curves to those obtained with human monocyte-derived macrophages (MDM). As shown in Figure 2 the murine cell line RAW264.7 responds similar to MDM when exposed to MAF02 particles and therefore seems to be a proper model system. For the experiments presented in the following we used a maximal MAF02 concentration of 50 μg/ml (15.6 μg/cm^2^) which did not affect viability after 24 h but induced a moderate increase of intracellular ROS levels after 3 h.

### Fly ash exposure induces liberation of arachidonic acid in RAW264.7 cells and human MDM

**Figure 3:**
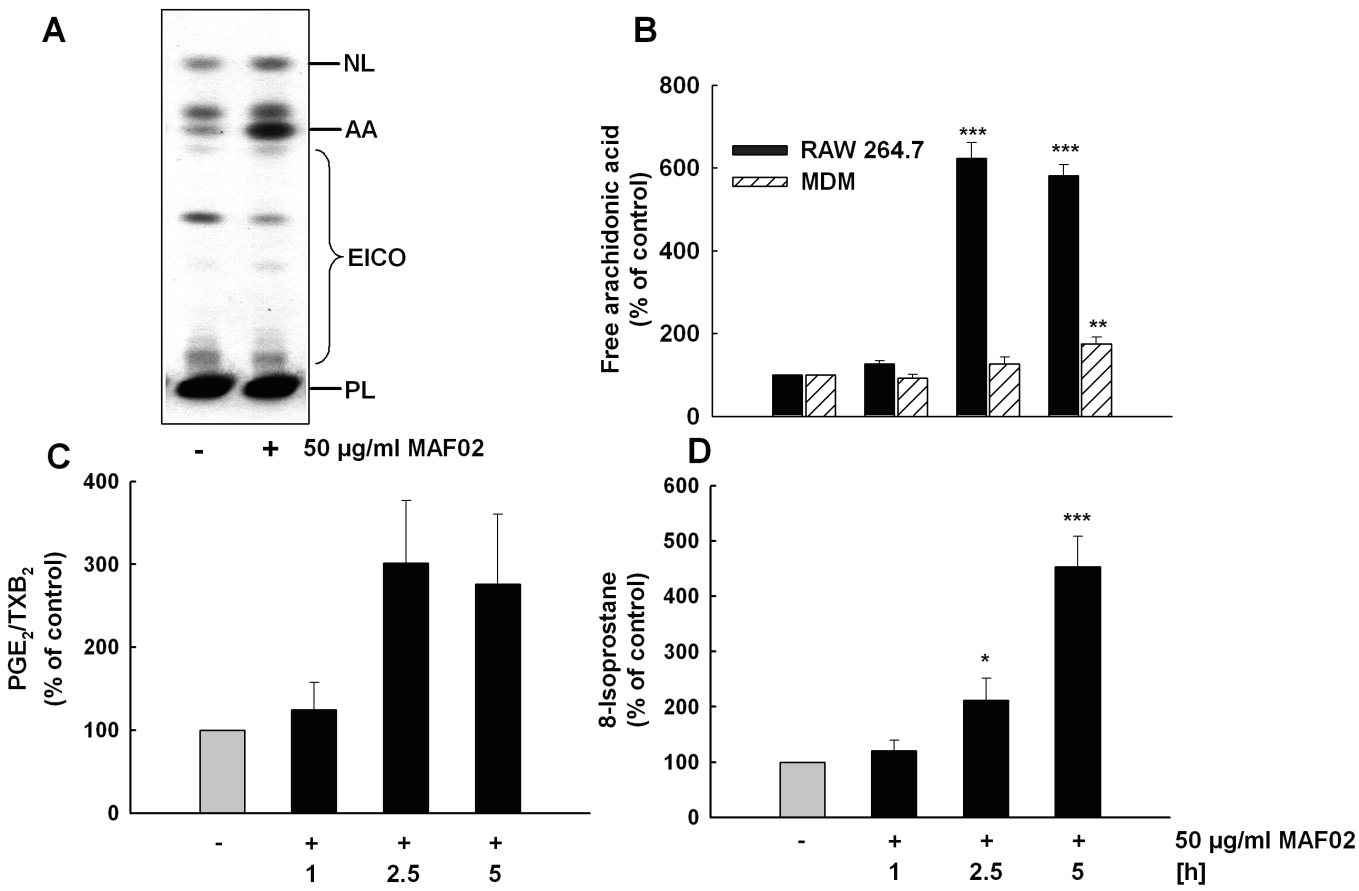
**Exposure to fly ash particles induces liberation of AA and release of PGE_2 _and 8-isoprostane from macrophages**. RAW264.7 macrophages and human MDM, pre-labelled with [^14^C]arachidonic acid, were incubated for 1, 2.5, and 5 h with MAF02 particles at 50 μg/ml (13.2 μg/cm^2^). After lipid extraction, AA and lipid mediators PGE_2_/TXB_2 _were separated by TLC (A, example of RAW264.7 cells treated for 2.5 h), and pixel images generated by phosphoimaging were quantified by OptiQuant^® ^software. Semiquantitative data on AA (B) and PGE_2_/TXB_2 _(C, only RAW264.7) liberation based on electronic autoradiographic values are expressed as percentage of control cells. (D) RAW264.7 macrophages were incubated for 1, 2.5, and 5 h with fly ash particles at 50 μg/ml (10.4 μg/cm^2^) and 8-isoprostane in the medium was determined by EIA. Results are presented as the mean of three independent experiments and error bars represent the standard error of the mean. Statistically significant differences from untreated control cells (*p < 0.05, **p < 0.01, *** p < 0.001). NL, neutral lipids; AA, free arachidonic acid; EICO, eicosanoids; PL, phospholipids.

AA mobilization in RAW264.7 macrophages was dependent on dose and time as already shown previously [17]. To provide further evidence that RAW264.7 cells serve as a reliable and accurate model system to investigate particle-induced cellular inflammatory processes, we compared the influence of MAF02 on AA mobilization to primary human MDMs. As shown in Figure 3B, we observed a moderate increase of AA mobilization in MDM. However, while a dose of 50 μg/ml (13.2 μg/cm^2^) MAF02 particles induced a 6-fold increase above the basal level of free AA in RAW264.7 cells after 5 h, a 1.8-fold increase was observed in MDM under the same exposure conditions. The lower potential of MAF02-mediated AA mobilization in MDM in comparison to RAW264.7 macrophages might be explained by a decreased expression of the key enzyme of AA mobilization, the cPLA_2_, in the primary cells compared to the mouse cell line (data not shown).

### Fly ash exposure induces the release of the AA-derivatives PGE_2_/TXB_2 _and 8-isoprostane

Free AA can be metabolized into prostaglandins and thromboxanes by oxygenation via the cyclooxygenase (COX) pathway. Two isoforms of the COX have been identified, COX-1, which is constitutively expressed, and COX-2, which can be induced by various inflammatory and proliferative stimuli [35,36]. Additionally, AA incorporated into phospholipids can be non-enzymatically converted to isoprostanes by free radical-initiated peroxidation.

The effect of MAF02 particles on activation of the AA pathway prompted us to investigate whether the increased liberation of AA is accompanied by elevated levels of AA-metabolites. Previously we could show an induction of COX-2 by MAF02 particles, whereas no increase in COX-1 protein was observed (data not shown). As shown in Figure 3C, a low dose of MAF02 at 50 μg/ml (10.4 μg/cm^2^) induced a release of PGE_2_/TXB_2 _from RAW264.7 cells in a time-dependent manner up to 5 hours of exposure. Similarly, exposure to the same concentrations and time periods led to the release of 8-isoprostane (Figure 3D), one of the most abundant isoprostanes, which serves as reliable biomarker of oxidative stress.

### Fly ash-induced liberation of arachidonic acid is regulated by cytosolic phospholipase A_2_

**Figure 4:**
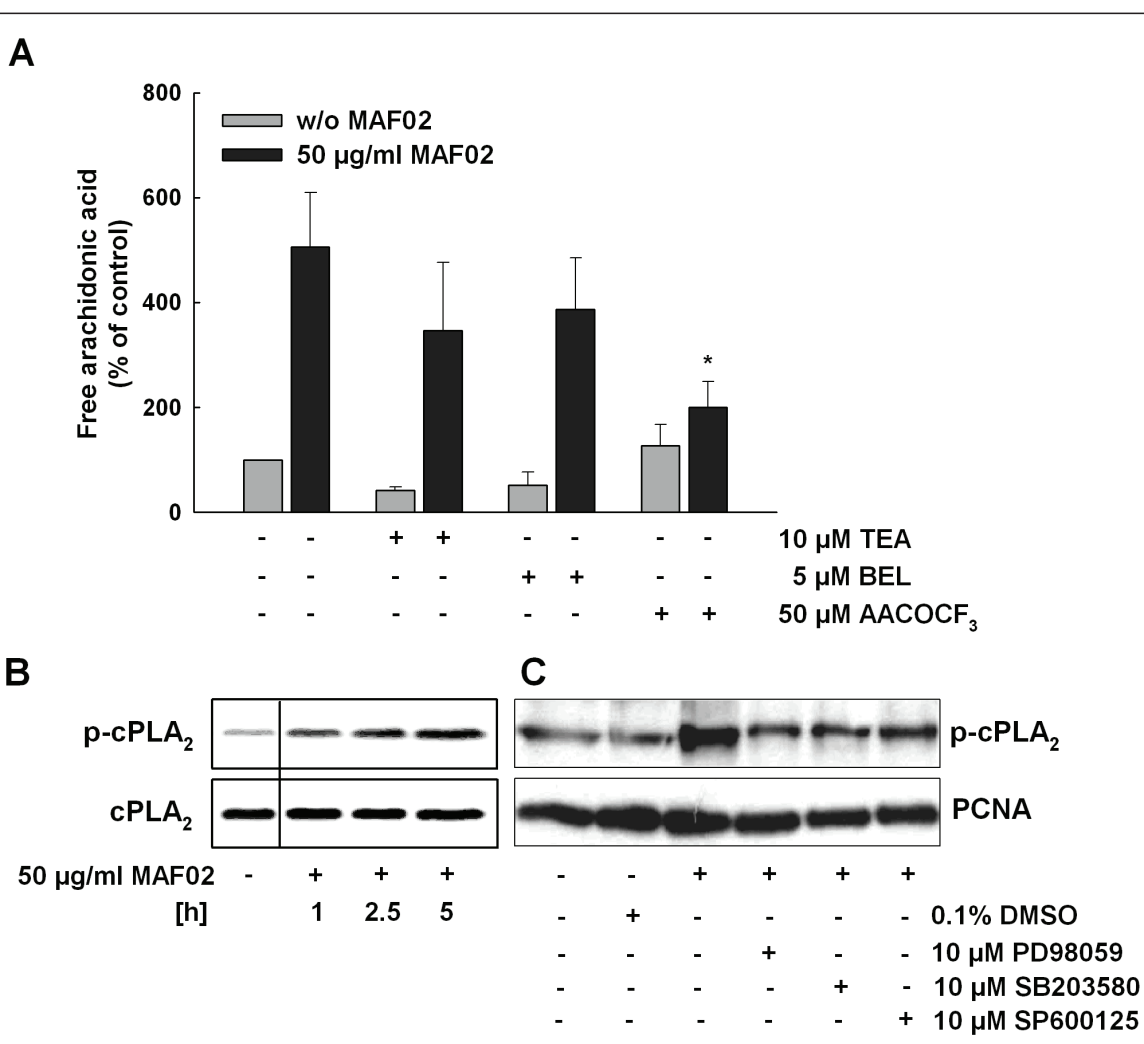
**MAF02-induced liberation of arachidonic acid is dependent on cPLA_2_**. (A) RAW264.7 macrophages, pre-labelled with [^14^C]arachidonic acid, were incubated with AACOCF_3 _(inhibitor of cPLA_2_), BEL (inhibitor of iPLA_2_) or TEA (inhibitor of sPLA_2_), and subsequently treated with 50 μg/ml MAF02 particles (13.2 μg/cm^2^) for 2.5 hours. After lipid extraction, the free arachidonic acid and its metabolites were separated by TLC, visualized by autoradiography and the optical density of the bands were analyzed by Odyssey^® ^software. [^14^C]arachidonic acid liberation data are expressed as percentage of untreated control cells (100%). Results are shown as the mean ± s.e.m. of three independent experiments. *Significantly decreased compared to 50 μg/ml (13.2 μg/cm^2^) MAF02-treated control cells (*p < 0.05). (B) RAW264.7 macrophages were exposed to 50 μg/ml MAF02 particles (15.6 μg/m^2^) for 1, 2.5 and 5 hours and whole cell lysates were analyzed for the phosphorylated cPLA_2 _protein by Western blotting. The respective non-phosphorylated protein was used as loading control. (C) RAW264.7 macrophages were pre-incubated with MAPK inhibitors PD98059, SB203580 and SP600125 (each 10 μM) and then exposed to 50 μg/ml MAF02 particles (15.6 μg/m^2^) for 5 hours. Whole cell lysates were analyzed for the phosphorylated cPLA_2 _protein, COX-2 and PCNA as loading control. The shown blots are representative of three independent experiments.

The AA, normally incorporated at the sn-2 position of phospholipids, can be released by activated phospholipases A_2 _(PLA_2_). The protein superfamily of phospholipases A_2 _includes the secretory (sPLA_2_), the cytosolic (cPLA_2_), and the Ca^2+^-independent PLA_2 _(iPLA_2_). Hence the next question was which of the PLA_2 _are involved in the MAF02-induced AA mobilization. To analyze the influence of these PLA_2 _on MAF02-induced AA liberation different PLA_2 _inhibitors were used. The RAW264.7 macrophages were preincubated with certain concentrations of specific inhibitors for the respective PLA_2 _isoform for 30 minutes and then treated with 50 μg/ml (13.2 μg/cm^2^) MAF02 particles over a period of 2.5 hours (Figure 4A).

Thioetheramide-phosphatidylcholine (TEA-PC), a specific inhibitor of the sPLA_2 _is an analogue of phosphatidylcholine, containing a thioether at the sn-1 position and an amide at the sn-2 position. Thereby it functions as a competitive, reversible inhibitor of sPLA_2 _[37]. In the experiment the preincubation with 10 μM TEA-PC reduced the particle-induced AA mobilization to approximately 60%, which however was not significant.

Treatment with 50 μM arachidonyltrifluoromethyl-ketone (AACOCF_3_) most effectively inhibited the MAF02-induced liberation of arachidonic acid down to 25%. AACOCF_3 _is a plasma membrane substrate analogue of arachidonic acid [38], which blocks the catalytic center of the cPLA_2 _by binding to serine reversibly. At higher concentrations it can also inhibit the iPLA_2 _while sPLA_2 _is not affected [39].

In order to investigate whether the iPLA_2 _also plays a role in the mobilization of AA in RAW264.7 macrophages, bromoenol lactone (BEL) as a selective, irreversible inhibitor of this enzyme was used [39]. As shown in Figure 4A the preincubation of the cells with 5 μM BEL had no significant influence on the particle-induced liberation of AA in comparison to macrophages, which were only treated with fly ash particles. Therefore it could be excluded that the iPLA_2 _was involved in the MAF02-triggered mobilization of AA. This also demonstrates that the reduction of the MAF02-induced AA mobilization by AACOCF_3 _was only due to inhibition of the cPLA_2 _but not of the iPLA_2_.

In summary, the cytosolic PLA_2 _and to a minor extent the secretory PLA_2 _but not the calcium-independent PLA_2 _are involved in the process of MAF02-induced liberation of arachidonic acid.

The cPLA_2 _activation is mediated by its phosphorylation [40]. Therefore, we analyzed the phosphorylation status of cPLA_2 _in response to MAF02 fly ash. Indeed, we detected an increase of the phosphorylated form of cPLA_2 _in RAW264.7 macrophages after treatment with MAF02 particles at 50 μg/ml (15.6 μg/cm^2^) by Western blot analysis using phospho-specific antibodies. In correlation with the inhibitor studies the degree of cPLA_2 _phosphorylation increased in a time-dependent manner from one hour to five hours of exposure and thus paralleled the enhanced AA liberation (Figure 4B).

We furthermore investigated by specific inhibitors if the MAPKs ERK1/2, JNK1/2 and p38 contribute to the MAF02-induced cPLA_2 _phosphorylation. As shown in Figure 4C, cPLA_2 _phosphorylation was reduced by PD98059, an inhibitor of the upstream kinase MEK1/2 which is responsible for ERK1/2 activation and by SB203580, an inhibitor of p38 activation. However, SP600125, an inhibitor of JNK1/2 activation, less efficiently blocked cPLA_2 _phosphorylation. A similar profile was observed for the induction of MAF02-induced expression of COX-2, which was prevented by the MEK1/2 and the p38 inhibitor but not by the JNK1/2 inhibitor.

### MAP kinases contribute to MAF02-induced AA mobilization

**Figure 5:**
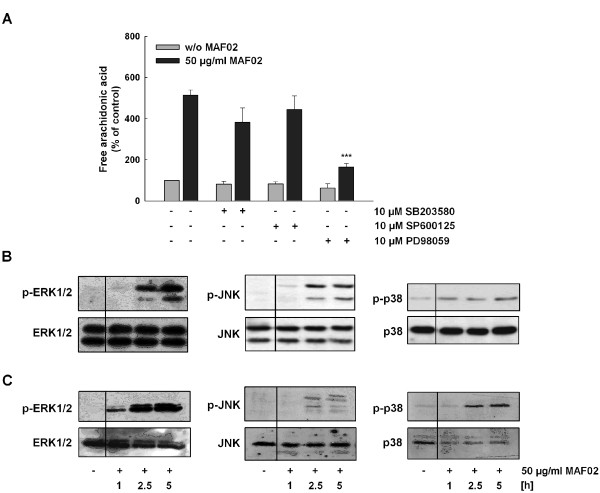
**MAF02-induced mobilization of AA depends on ERK1/2 and MAPKs are activated in RAW264.7 macrophages as well as in human MDM**. (A) RAW264.7 cells labelled with [^14^C]arachidonic acid were pre-incubated for 30 min with the indicated kinase inhibitors and subsequently exposed to 50 μg/ml MAF02 particles (12.6 μg/cm^2^) for 2.5 h. After lipid extraction, free AA was separated by TLC, visualized by autoradiography, and the optical density of the bands were analyzed by OptiQuant^® ^and Odyssey^® ^software. [^14^C]arachidonic acid mobilization data are expressed as a percentage of the values for control cells (100% values). Results are the mean ± s.e.m. of three independent experiments (***p < 0.001 compared to 50 μg/ml MAF02-treated cells). RAW264.7 macrophages (B) and human MDM (C) were treated with 50 μg/ml MAF02 particles (15.6 μg/m^2^) for 1, 2.5 and 5 hours and whole cell lysates were analyzed for the target proteins by Western blotting. The respective non-phosphorylated proteins are used as loading control.

Using phospho-specific antibodies we detected increased phosphorylation of ERK1/2 and JNK1/2 after treatment with fly ash in dependence of time, reaching its maximum after 5 hours. On the other hand, p38 MAPK was only weakly phosphorylated after treatment of RAW264.7 cells with MAF02 particles (Figure 5B). Only inhibition of the ERK1/2 pathway with PD98059 lead to a significant reduction of AA mobilization (Figure 5A) confirming the contribution of ERK1/2 in activation of the cPLA_2 _already shown in Figure 4C. The p38 and JNK1/2 inhibitors only moderately reduced the fly ash-mediated liberation of AA but not significantly.

Again, the primary human MDM showed a similar time-dependent increase of ERK1/2 and JNK1/2 phosphorylation as well as an even stronger activation of p38 MAPK (Figure 5C).

### NAC reduces fly ash-induced signalling and AA mobilization

**Figure 6:**
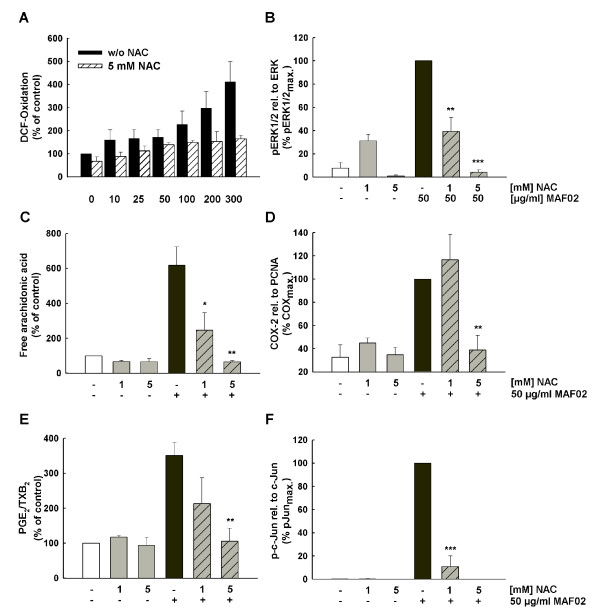
**NAC inhibits fly ash-induced formation of ROS, mobilization of AA, phosphorylation of ERK1/2, JNK, c-Jun and COX-2 expression**. (A) Adherent H_2_DCF-loaded RAW264.7 cells were pre-incubated with 5 mM NAC and further incubated with 10, 25, 50, 100, 200 and 300 μg/ml fly ash particles (6.3, 12.6, 31.3, 62.5, 125, 188 μg/cm^2^) for 2.5 hours. Values are expressed as a percentage of the untreated controls. For analysis of ERK1/2 (B), c-Jun (F) phosphorylation, and COX-2 expression (D), non-labelled cells were pre-incubated with NAC at 1 and/or 5 mM and further treated with 50 μg/ml MAF02 particles (15.6 μg/m^2^) for 2.5 hours. Whole cell lysates were analyzed by Western blotting. OD-band intensities of COX-2 and phospho-proteins, analyzed by Odyssey^® ^software, were normalized to the respective loading control protein and expressed in relation to the maximum band intensity of the MAF02-treated sample (100%). To detect mobilization of AA and its metabolites (C, E), RAW264.7 macrophages labelled with [^14^C]arachidonic acid were pre-incubated with the antioxidant NAC at 1 or 5 mM and subsequently exposed with 50 μg/ml MAF02 particles (13.2 μg/m^2^) for 2.5 hours. After lipid extraction, the free arachidonic acid and its metabolites were separated by TLC, visualized by autoradiography and analyzed by OptiQuant^® ^software. Data on AA liberation are expressed as percentage of control cells (100% values). Results are presented as the mean ± s.e.m. of three independent experiments (* p < 0.05, **p < 0.01, ***p < 0.001 compared to 50 μg/ml MAF02-treated cells).

To explore the involvement of ROS in MAF02-mediated AA mobilization, RAW264.7 macrophages were pre-incubated for 30 min with the antioxidant NAC prior to fly ash exposure. We observed in RAW264.7 macrophages that fly ash-induced production of ROS (Figure 6A), phosphorylation of ERK1/2 (Figure 6B), mobilization of AA (Figure 6C) as well as COX-2 protein expression (Figure 6D) together with the release of PGE_2_/TXB_2 _(Figure 6E) were inhibited by 5 mM NAC nearly completely, while 1 mM NAC had only a weak effect on the induction of these processes. In contrary, fly ash-induced phosphorylation of c-Jun (Figure 6F) together with activation of JNK1/2 (data not shown) were inhibited almost completely with only 1 mM NAC while AA liberation is still induced and only totally blocked at 5 mM. Together with the fact that inhibition of JNK did not reduce AA mobilization as shown above we conclude that JNK signalling seems not to be involved in mobilization of AA and its metabolites. The observed fly ash-induced activation of JNK1/2 and c-Jun may trigger other endpoints, which were not further studied. Thus, MAF02 fly ash exerts its effects via the formation of ROS, which is a prerequisite for the activation of the MAPK pathways and mobilization of AA via activation of cPLA_2_, and its subsequent conversion by COX-2 to the lipid mediators PGE_2_/TXB_2_.

### The water-insoluble fraction of MAF02 is responsible for AA mobilization

**Figure 7:**
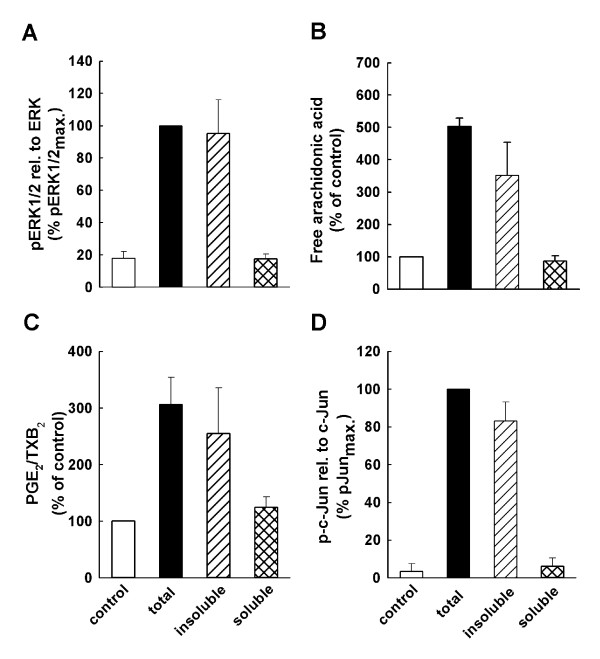
**The water-insoluble fraction of MAF02 is responsible for AA mobilization and activation of ERK1/2 and c-Jun**. For analysis of ERK1/2 (A), and c-Jun (D) phosphorylation non-labelled cells were exposed with 50 μg/ml (15.6 μg/m^2^) of the total, water-soluble and water-insoluble fraction of fly ash particles for 2.5 hours. Whole cell lysates were analyzed by Western blotting. OD-band intensities of phosphorylated proteins, analyzed by Odyssey^® ^software, were normalized to the respective loading control protein and expressed in relation to the maximum band intensity of the MAF02-treated sample (100%). (B, C) RAW264.7 cells labelled with [^14^C]arachidonic acid were treated with 50 μg/ml (13.2 μg/m^2^) of the total, water-insoluble and water-soluble fraction of fly ash particles for 2.5 hours. After lipid extraction, the free arachidonic acid and its metabolites were separated by TLC, visualized by autoradiography and analyzed by OptiQuant^® ^and Odyssey^®^software. Data on AA liberation are expressed as percentage of control cells (100% values). Results are presented as the mean ± s.e.m. of three independent experiments.

To examine whether the water-soluble or insoluble fraction of MAF02 are involved in the fly ash-induced activation of the AA cascade, RAW264.7 macrophages were exposed for 2.5 h to 50 μg/ml (13.2 to 15.6 μg/cm^2^) of the total, water-insoluble (pellet) or water-soluble (supernatant) fraction of fly ash particles (Figure 7). We observed that exposure of macrophages to the water-insoluble pellet fraction of MAF02 particles induced a marked AA and PGE_2_/TXB_2 _mobilization similar to the liberation of AA and its metabolites in cells treated with the total fraction of fly ash particles. In contrast, the soluble fraction of MAF02 did not induce any significant increase in AA and PGE_2_/TXB_2 _release, indicating that the water-insoluble fraction of MAF02 causes the cellular particle-induced effects. In addition, while exposure of RAW264.7 cells to the pellet fraction of fly ash resulted in an enhanced phosphorylation of ERK1/2 and of the transcription factor c-Jun, the supernatant fraction did not activate ERK1/2 and c-Jun. These results indicate that the metals which are still incorporated in the glass-like matrix of the water-insoluble fraction and probably become soluble intracellularly due to the low pH within lysosomes are responsible for the detected cellular events.

### DFO reduces fly ash-induced signalling and AA mobilization

**Figure 8:**
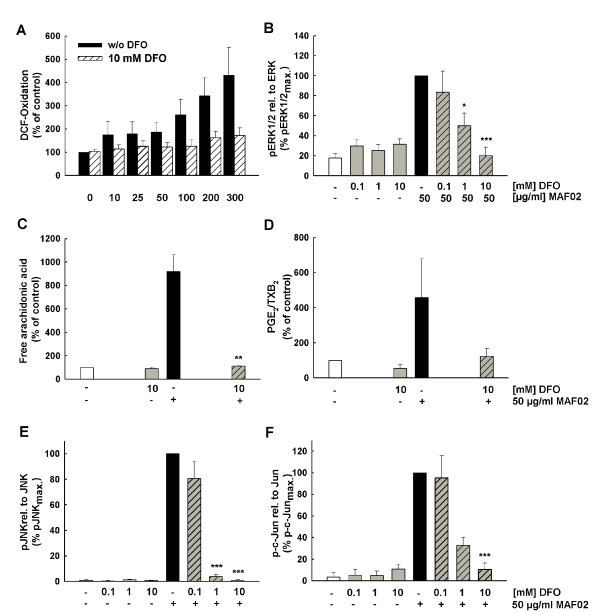
**DFO inhibits the MAF02-induced formation of ROS, mobilization of AA, PGE_2_/TXB_2 _and phosphorylation of ERK1/2**. (A) Adherent H_2_DCF-loaded RAW264.7 cells were incubated with DFO before treatment with 10, 25, 50, 100, 200 and 300 μg/ml MAF02 particles (6.3, 12.6, 31.3, 62.5, 125, 188 μg/cm^2^) for 2.5 hours. Values are expressed as a percentage of the untreated controls. For analysis of ERK1/2 (B), JNK (E), and c-Jun phosphorylation non-labelled cells were pre-incubated with various concentrations of DFO and subsequently exposed with 50 μg/ml MAF02 particles (15.6 μg/m^2^) for 2.5 hours. Whole cell lysates were analyzed by Western blotting. OD-band intensities of phosphorylated proteins, analyzed by Odyssey^® ^software, were normalized to the respective loading control protein and expressed in relation to the maximum band intensity of the MAF02-treated sample (100%). [^14^C]arachidonic acid-labelled RAW264.7 macrophages were treated with DFO before exposure with 50 μg/ml MAF02 particles (13.2 μg/m^2^) for 2.5 hours. After lipid extraction, the free arachidonic acid and its metabolites were separated by TLC, visualized by autoradiography and analyzed by OptiQuant^® ^software. Data on AA (C) and PGE_2_/TXB_2 _(D) liberation are expressed as percentage of control cells (100% values). Results are presented as the mean ± s.e.m. of three independent experiments (* p < 0.05, **p < 0.01, p*** < 0.001 compared to 50 μg/ml MAF02-treated cells).

Several studies have demonstrated that redox-active metals in particular iron and zinc of ambient particulate matter can induce an increased production of ROS which may lead to oxidative stress. The water-insoluble pellet fraction of MAF02 particles consists of appreciable amounts of zinc and iron which could cause the generation of ROS by e.g. an iron-mediated Fenton-like reaction. In order to investigate an association between the metal content of MAF02 particles and activation of the AA cascade the metal chelator DFO was added to the RAW264.7 macrophages prior to stimulation with fly ash particles for 2.5 h. Pre-treatment of macrophages with DFO resulted in a decreased particle-induced formation of ROS (Figure 8A) and prevented the subsequent phosphorylation of the ERK1/2 MAPK (Figure 8B), together with the liberation of AA (Figure 8C) and its metabolization to PGE_2_/TXB_2 _(Figure 8D). Phosphorylation of JNK and c-Jun was also inhibited by DFO (Figure 8E-F). The clear inhibition of these effects by DFO as well as the observation that only the water-insoluble fraction is responsible for the effect (Figure 7) demonstrates that the MAF02 particles induce activation of the AA signalling pathway by metals which are included in the water-insoluble matrix, but which may become bioavailable inside the cell. The effects of DFO on the AA pathway support the hypothesis that activation of the AA cascade by fly ash particles depends on metal-mediated ROS formation.

## Discussion

Many studies detected multiple effects of PM on e.g. inflammatory pathways in different cell types but the challenge is to identify the key mechanisms which initiate all these pleiotropic downstream responses. Here we pinpoint the critical role of metals in PM initiated inflammatory signalling in macrophages. In the following we discuss the underlying mechanisms of signal transduction and how this relates to findings observed with other types of PM.

Our studies focused on the MAF02-induced inflammatory processes with special emphasis on the regulation of the AA metabolism. AA liberation and its metabolization to lipid mediators are relevant in initiation, maintenance and resolution of inflammation and therefore play a significant role in chronic inflammation. Furthermore, disturbed regulation of AA metabolism may contribute to cancer diseases in particular those of the lung [20-22].

The fly ash particles induced a strong mobilization of AA at non-cytotoxic concentrations. For similar metal-laden combustion-derived particles such as residual oil fly ash (ROFA) the influence of lipid mediators in mediating pulmonary toxicity has been shown *in vivo *and *in vitro *[41] pointing to deregulated lipid mediators as a central toxicity pathway.

Human monocyte-derived macrophages (MDM) were tested for their response to MAF02, however we only observed a 1.8-fold increase of AA liberation after 5 hours exposure in comparison to a 6-fold increase in RAW264.7 macrophages. The lower expression of cPLA_2 _in MDM compared to RAW264.7 cells may be a reason for their reduced potential to induce AA mobilization (data not shown). Alternatively, human macrophages might be less susceptible to PM-induced AA liberation, a possibility which needs to be further investigated. Although the AA mobilization after MAF02 treatment was not as pronounced as in RAW264.7 macrophages, the increase in ROS and MAPK activity as well as the reduction in viability after exposure to MAF02 was quite comparable to the effects observed in RAW264.7 cells. Therefore, it seems that the signalling cascades induced by fly ash particles in RAW264.7 cells are conserved in primary human macrophages.

The mobilized arachidonic acid may be further metabolized by cyclooxygenases (COX) to biologically active mediators such as leukotrienes, prostaglandins and thromboxanes, which play a role during inflammatory processes. Here we demonstrate that MAF02-induced COX-2 is also catalytically active since the level of the metabolites PGE_2_/TXB_2 _in the supernatant increased in a time-dependent manner. Particle-induced induction of COX-2 as well as release of PGE_2 _by immune-competent cells has also been shown in other studies [18,42-44]. The consequences of increased PGE_2 _release cannot be predicted directly since it regulates pro-inflammatory as well as anti-inflammatory effects. The action of PGE_2 _is dependent on the presence or absence of PGE_2 _receptors EP1 - EP4 on the target cells. PGE_2 _has therefore the ability to switch from pro-inflammatory to anti-inflammatory mechanisms. Anti-inflammatory effects have been shown in human monocytes where PGE_2 _down-regulated TNF-α induced expression of ICAM-1 by interaction with EP2 and EP4 [45]. In contrary, interaction with EP1 and EP3 induced proinflammatory effects. Beck-Speier *et al*. [46] suggest, that PGE_2 _released due to fine TiO_2 _particles with low surface area may act anti-inflammatory in that it down-regulates particle-induced inflammatory effects. However, PGE_2 _released due to ultrafine TiO_2 _particles with large surface area may act proinflammatory since the inflammatory mediator leukotriene B_4 _as well as release of 8-isoprostane were also induced. The basis for these size-dependent differences remains to be clarified.

MAF02 exposure induced enhanced ROS levels as measured by the DCF assay. However, as the DCF assay sometimes produces false positive results [47] we analysed previously additional markers of oxidative stress e.g. HO-1 induction and increase of GSH synthesis [13,17]. In addition, in the present study we also detected a significant release of 8-isoprostane, which further demonstrates the high oxidative potential of the particles on cell membranes. 8-isoprostane, however, has numerous biologic effects, e.g. it is a potent vasoconstrictor, causes contraction of bronchial smooth muscle and induces exudation in the airways [48,49]. Therefore, the enhanced formation of disease-related 8-isoprostane can contribute to the adverse health effects of particulate matter.

In most cases, the cause of AA mobilization and generation of AA-derived lipid mediators is the activation of phospholipase A_2 _which catalyzes the deacylation of AA from the sn-2 position of membrane glycerophospholipids. In this study we demonstrated that the MAF02-induced AA mobilization is mainly mediated by the Ca^2+^-dependent cPLA_2_, not by iPLA_2 _and sPLA_2_. This is supported by the finding that MAF02-induced AA mobilization is inhibited by the intracellular calcium chelator BAPTA/AM [17] as well as by the extracellular calcium chelator EGTA (data not shown). An elevated intracellular Ca^2+ ^concentration is necessary for the translocation of the activated cPLA_2 _to its target structure in perinuclear membranes [50]. Stone and colleagues [51] observed an increase of the intracellular Ca^2+^-concentration in the human Mono Mac 6 cell line after exposure to ultrafine carbon black particles, which could also be inhibited by EGTA as well as by the calcium channel blocker verapamil. The authors suggest that ROS triggers an opening of the Ca^2+^-channels which lead to a flux from the extracellular compartment into the cytosol [51,52].

In MAF02-treated cells cPLA_2 _was phosphorylated which is required for activation of the enzyme. The time course of phosphorylation was in accordance with the MAF02-induced AA mobilization and could be reduced by inhibition of the ERK1/2 and p38 MAPKs. Activation of cPLA_2 _by phosphorylation via the ERK1/2 and the p38 MAPK signalling pathways has already been described [50]. Using phospho-specific antibodies we found in this study that ERK1/2 and JNK1/2 were phosphorylated after treatment of RAW264.7 macrophages and MDM with MAF02 particles with similar kinetic compared to the mobilization of AA whereas p38 MAPK was only weakly phosphorylated. Thus MAPKs activity is not only required to activate the cPLA_2 _and mobilize AA but is also induced in response to MAF02. Similar results were found in primary canine alveolar macrophages which were exposed to diesel exhaust particles. Inhibitor studies indicated an involvement of ERK1/2 but not of p38 MAPK in the DEP-induced mobilization of AA and synthesis of its metabolites PGE_2 _and LTB_4 _[18].

The results so far indicate an involvement of ROS and oxidative stress in the cellular responses to the fly ash particles. To demonstrate involvement of ROS in the AA metabolism we used the antioxidant NAC, a general antioxidant but also a metal-binding agent [53]. NAC has been used as a tool for investigating the role of ROS in numerous biological and pathological processes [54]. We could show that pre-treatment of RAW264.7 macrophages with 5 mM NAC resulted in significant inhibition of fly ash-induced phosphorylation of ERK1/2, mobilization of AA, and induced expression of COX-2. This clearly demonstrates a contribution of ROS and possibly metals in these mechanisms. While pre-incubation of the cells with 1 mM NAC had no or only a weak effect on these responses, surprisingly the MAF02-induced phosphorylation of JNK1/2 as well as of c-Jun was completely inhibited at this low NAC concentration. This means that the activation of the JNK1/2 signalling pathway is, although ROS dependent, probably not involved in the mechanisms of MAF02-induced mobilization of AA, and expression of COX-2, at least in RAW264.7 macrophages. In accordance with this hypothesis, the specific inhibitor of the JNK1/2 pathway SP600125 did not prevent AA mobilization and COX induction by MAF02 thus demonstrating that indeed the JNK cascade is not involved in this response.

The mechanisms by which endogenously produced ROS or metals activate MAP kinases are not well defined. One possible explanation is the regulation of the MAPK activity by protein phosphatases, which are known to be sensitive to ROS and metals because of their cysteinyl thiol groups in their active site [11,55,56].

The fly ash used in this study has a high content of water-soluble material (61% by weight) which has been shown to be biologically inactive in our assays. This is contrary to other studies with complex material such as ambient particulate matter where water-soluble metals were shown to be associated with induction of ROS, oxidative stress and pro-inflammatory effects [33]. Although transition metals such as Fe and Zn are significant components of the MAF02 fly ash, the water-soluble portion of these metals may be too low-concentrated to catalyze the formation of ROS via a Fenton-like reaction [32,57-59]. The insoluble fraction still contains metals in a glass-like matrix and those metals on the surface may become soluble intracellularly and thereby responsible for the observed effects. The metal chelator DFO prevented entirely ROS formation and downstream signalling suggesting a critical role of metals in the inflammatory response triggered by MAF02. Thus, in the absence of transition metals the remaining particulate fraction is inactive and does not trigger inflammation mediated by AA. The relevance of biologically available transition metals in particular iron in inducing adverse effects has been demonstrated for air pollution particles [60], asbestos [61], and carbon nanotubes [62].

**Figure 9:**
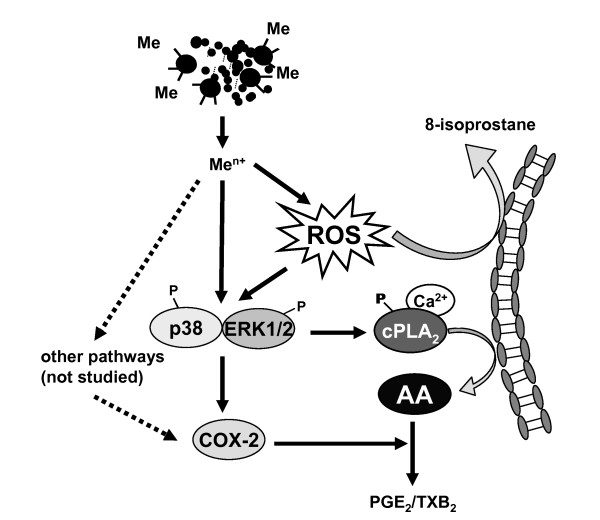
**Scheme of the MAF02-induced activation of the arachidonic acid cascade**. The MAF02 particles are taken up by RAW264.7 macrophages and release metal ions (Me^n+^) inside the cells which induces formation of reactive oxygen species (ROS). Release of 8-isoprostane indicates the occurrence of oxidative stress. Oxidative stress in turn leads to activation of the ERK1/2 and p38 pathways. The ERK1/2 pathway may be activated by interfering of the particles with membrane proteins. On the other hand, enhanced MAPK activation can also occur by inhibition of metal- and redox-sensitive phosphatases, which dephosphorylate and inactivate MAPK. Activation of ERK1/2 and p38 leads to phosphorylation and activation of cPLA_2 _(cytosolic phospholipase A_2_). The increase of the intracellular calcium level induces cPLA_2 _translocation to the perinuclear membranes where it catalyzes the mobilization of AA. On the other hand, it is possible that ROS inhibit the reacylation of AA, which would also lead to an enhanced level of free AA in the cytosol. Free AA is converted by COX-2 (cyclooxygenase-2) and prostaglandin synthases to PGE_2_/TXB_2 _(prostaglandin E_2_/thromboxane B_2_). Increased transcription of COX-2 requires activation of ERK1/2 and p38, but may also involve other pathways e.g. NF-κB.

On the basis of the data obtained from the present and the previous study we suggest the following mechanism (Figure 9):

The MAF02 particles are taken up by RAW264.7 macrophages and induce a metal-mediated generation of reactive oxygen species. Furthermore, release of 8-isoprostane, increased synthesis of glutathione and induction of heme oxygenase-1 [17] indicate significant oxidative stress due to particle exposure resulting in an anti-oxidative cellular response. Oxidative stress leads to activation of the ERK1/2 pathway including MEK1/2, JNK1/2 and p38 MAP kinases. The ERK1/2 pathway may be activated by interference of the particles with membrane proteins such as growth factor receptors. Growth factor receptors and MAPK can also be stimulated by inhibition of phosphatases, which are metal- and redox-sensitive enzymes.

Active ERK1/2 and p38 leads to phosphorylation and activation of cPLA_2_. The increase of the intracellular calcium level induces the cPLA_2 _to translocate to the perinuclear membranes where it catalyzes the mobilization of AA. In addition, it is possible that ROS inhibit the reacylation of AA, which would also lead to an enhanced level of free AA in the cytosol. Free AA serves as substrate for COX-2 and prostaglandin synthases leading to the release of PGE_2_. Increased transcription of COX-2 requires activation of ERK1/2 and p38, but may also involve other pathways e.g. NF-κB [63].

## Conclusions

In summary, the results of the present study suggest, that exposure of macrophages to fly ash particles promotes inflammation by liberation of arachidonic acid and metabolization to related products such as PGE_2_/TXB_2 _via metal-induced formation of ROS and induction of cellular oxidative stress.

The fly ash-induced processes such as formation of ROS, activation of ERK1/2, JNK1/2 and p38 MAPK pathways were also observed in primary human MDM, however mobilization of AA was less severe possibly because of the incomplete differentiation to macrophages.

The postulated mechanism may therefore also be relevant in humans, where it may contribute to lung diseases such as chronic inflammation after acute or chronic exposure to fine and ultrafine particulate matter.

## Methods

### Materials

Cell culture medium and supplements, Hank's balanced salt solution (HBSS) and 2',7'-dichlorodihydrofluorescein-diacetate (H_2_DCF-DA) were obtained from Invitrogen (Karlsruhe, Germany). Accutase was from PAA (Cölbe, Germany). The WST-1 reagent was from Roche (Mannheim, Germany) and the SDS-PAGE supplies were from Carl Roth (Karlsruhe, Germany). [1-^14^C]arachidonic acid (2.07 GBq/mmol) and ECL reagents were purchased from GE Healthcare (Freiburg, Germany). Chemicals for lipid extraction and chromatography were from VWR International (Bruchsal, Germany). The PLA_2 _inhibitors thioetheramide phosphatidylcholine (TEA-PC), arachidonyl trifluoromethyl ketone (AACOCF_3_), and bromoenol lactone (BEL) were from Cayman (Biozol, Eching, Germany). MAPK inhibitors, PD98059 (2'-amino-3'-methoxyflavone), SB203580 [4-(4-fluorophenyl)-2-(4-methylsulfinylphenyl)-5-(4-pyridyl)1H-imidazole], and SP600125 (1,9-pyrazoloanthrone) were purchased from Merck (Darmstadt, Germany). N-acetyl-cysteine (NAC), deferoxamine mesylate (DFO) and standard laboratory chemicals were supplied by Sigma-Aldrich (Taufkirchen, Germany). The human recombinant granulocyte-macrophage colony-stimulating factor (GM-CSF) was from VWR, Bruchsal, Germany.

Specific anti-phospho ERK1/2 (Tyr^202^/Tyr^204^), anti-phospho p38 (Tyr^180^/Tyr^182^), anti-phospho JNK1/2 (Tyr^183^/Tyr^185^), anti-phospho c-Jun, anti-phospho MEK1/2, anti-phospho cPLA_2 _(Ser^505^), and anti-c-Jun were obtained from Cell Signaling (New England Biolabs, Frankfurt, Germany). Anti-ERK1/2, anti-p38, anti-JNK, anti-cPLA_2_, anti-LaminB, and anti-PCNA were from Santa Cruz (Heidelberg, Germany). Anti-COX-1 and anti-COX-2 were from Biozol (Eching, Germany). Horseradish peroxidase (HRP)-conjugated secondary anti-rabbit antibodies were from GE Healthcare, Braunschweig, Germany and anti-mouse antibodies from DAKO (Hamburg, Germany). IRDye700 or IRDye800 coupled secondary antibodies were obtained from Biomol (Hamburg, Germany).

### Particles

The fly ash MAF02 originates from a municipal waste incinerator facility and was collected in 2002 by electrostatic precipitation in the exhaust gas cleaning system. Subsequently, the powder was size-fractionated to remove particles > 20 μm. The remaining fine fraction (< 20 μm) has been used for the *in vitro *experiments. Analysis of the particle size distribution by scanning mobility particle sizing (SMPS, DMA 3071 with CPC 3022A, TSI Inc., Shoreview, USA) after resuspension in air showed that the number concentration was dominated by fine and ultrafine particles with a modal value of 165 nm [13]. Additional analyses using the light-scattering spectrometer PCS-2000 (Palas, Karlsruhe, Germany) and scanning electronic microscopy (SEM) of particles deposited on a Nuclepore™ Polycarbonate Membrane (pore size 0.4 μm, Whatman, Dassel, Germany) confirmed the low percentage of large agglomerates (Figure 1A-B).

Elemental analysis was performed with total particles as well as with the water-soluble and insoluble fraction. For the separation 1 g of particles was extracted two-fold with 25 ml of deionized water. The aqueous suspensions were probe sonified (Branson Sonifier 250, Heinemann, Schwäbisch-Gmünd, Germany) for 20 s (duty cycle 40, output control 4) and centrifuged at 1000 g for 30 min. The combined aqueous supernatants as well as the dried pellet were analyzed by Total Reflection X-ray Fluorescence Analysis (TRFA). As and Cd were measured by Inductively Coupled Plasma - Optical Emission Spectrometry (ICP-OES). The fraction of elemental carbon was determined by combustion of the material in a copper tube at 950°C and measuring the CO concentration.

For cell experiments stock suspensions of 10 mg/ml in culture medium without FBS were prepared freshly and dispersed by vortexing and probe sonication for 20 s. Working suspensions were prepared by further dilution in culture medium. The particles were tested for endotoxin content with the colorimetric Limulus Amebocyte Lysate (LAL) assay (Lonza, Basel, Switzerland). A particle suspension of 1 mg/ml in water was centrifuged for 10 min at 20,800 g and the supernatant was analyzed according to the instructions of the manufacturer. The result was below the lower limit of quantification of the test (< 0.1 EU/ml).

### Cell culture

The murine RAW264.7 macrophage cell line was obtained from the American Type Culture Collection (ATCC Rockville, MD, USA). The cells were cultured in DMEM supplemented with 10% fetal bovine serum (FBS), 100 U/ml penicillin and 100 μg/ml streptomycin in 5% CO_2 _at 37°C. They were passaged every 3 to 4 days by scraping off the cells from the culture plate. To investigate AA release and intracellular signalling pathways, cells were seeded onto 12-well plates at 3 × 10^5 ^cells/well or into 6-well plates at 2.5 × 10^6 ^cells/well. After adhering overnight, the cells were cultured for another day in serum-free medium prior to stimulation. All inhibitors were added 30 min prior to stimulation at the indicated concentrations.

### Generation of human monocyte-derived macrophages (MDM)

Human peripheral blood mononuclear cells (PBMC) were isolated from buffy coats derived from healthy donors (Städtisches Klinikum Karlsruhe) by Ficoll density gradient centrifugation (Ficoll-Paque™ Premium, GE Healthcare, Freiburg). For isolation of CD14-positive monocytes, the MACS magnetic separation technique (Miltenyi Biotec, Bergisch-Gladbach, Germany) was used according to the manufacturer's protocol. Briefly, 1 × 10^7 ^PBMC suspended in 80 μl of MACS buffer (phosphate-buffered saline (PBS) containing 0.5% BSA and 2 mM EDTA) were mixed with 20 μl of anti-CD14 microBeads and incubated at 4°C for 15 min. The cells were washed with 2 ml MACS buffer, resuspended in 500 μl fresh MACS buffer and loaded onto a separation column which was positioned in a MidiMACS magnet. Non-adherent cells were washed out and after removal from the magnet the CD14-positive cells were recovered from the column by pressing 5 ml of MACS buffer through the column.

The percentage of the purified monocytes was determined by flow cytometry (LSR II with CellQuest PRO software, Becton Dickinson, Heidelberg, Germany) using FITC-labeled anti-CD14 antibodies (My4-FITC, Coulter, Krefeld, Germany) and FITC-labelled anti-IgG2a antibodies (Miltenyi Biotec, Bergisch Gladbach, Germany) as isotype control. The cells were detected with an excitation wavelength of 488 nm and an emission wavelength at 530 nm. The purity of the preparation was determined to be 89.4 ± 1.2% (n = 4) of CD14-positive monocytes after the cell separation from different donors. The remaining 9 - 11% cells were lymphocytes, which were not further analyzed.

The isolated cells were resuspended in RPMI 1640 medium supplemented with 1% MEM non-essential amino acids, 1% pyruvate, 4% FBS, 100 U/ml penicillin, 100 μg/ml streptomycin, 2 mM glutamine and 2 ng/ml GM-CSF at 1 × 10^6 ^cells/ml and cultivated in 5% CO_2 _at 37°C for 10 days. Medium was changed every 3 to 4 days. Before the experiments, the MDM were detached from the surface using accutase and seeded into 96-well plates at 2 × 10^5 ^cells/well, into 12-well plates at 3 × 10^5 ^cells/well or into 6-well plates at 2.5 × 10^6 ^cells/well.

### Transmission electron microscopy (TEM)

To determine the time-dependent uptake of particles RAW264.7 macrophages were grown on Transwell^® ^inserts with polycarbonate membranes with 0.4 μm pores (Corning, Wiesbaden, Germany) and exposed to particle suspensions at 50 μg/ml (10.4 μg/cm^2^). Pieces of the membrane with the adherent cells were cut out and fixed in Karnovsky's fixant containing 2.5% glutaraldehyde (w/v) for 15 min. The membranes were washed with PBS, post-fixed in 1% (w/v) osmium tetroxide for 7 min and dehydrated in a graded series of ethanol (50, 70, 95, and 100%). Afterwards the membranes were embedded in EPON 812 (Polysciences, Eppelheim, Germany). The blocks were cutted into ultrathin sections using a UC6 ultramicrotome (Leica, Bensheim, Germany). Images were taken with a Zeiss EM 109T transmission electron microscope (Oberkochen, Germany).

### Viability assay

Cell viability was determined in 96-well plates by the WST-1 assay after treatment with the particles suspended in complete medium at the indicated concentrations for 24 h. The medium was replaced by 100 μl WST-1 reagent diluted 1:20 (v/v) with HBSS. After incubation at 37°C and 5% CO_2 _for 2 h the activity of mitochondrial dehydrogenases was detected with a microplate reader (Molecular Devices, Ismaning, Germany) at 450 nm.

### Intracellular ROS detection

Macrophages seeded in 96-well plates were treated with particles suspended in HBSS for 3 h at the doses indicated (Figure 2B). After washing with HBSS, the cells were loaded with 50 μM H_2_DCF-DA for 30 min. Subsequently, the cells were washed again with HBSS and the relative fluorescence units were determined at 485 nm excitation and 530 nm emission wavelengths using a fluorescence reader (MWG-Biotech AG, Ebersberg, Germany). Alternatively, the cells were loaded with 50 μM H_2_DCF-DA for 30 min prior to the particle incubation to improve sensitivity (Figure 8). After treatment with particles for 2.5 h, the cell layer was washed with HBSS and the relative fluorescence units were detected.

### Analysis of arachidonic acid and PGE_2_/TXB_2 _liberation

The determination of arachidonic acid and PGE_2_/TXB_2 _liberation in macrophages was performed as described before [17]. Briefly, after labelling with [1-^14^C]arachidonic acid the cells were treated with particles for the indicated periods of time in medium without FBS. The cellular lipids were extracted with a mixture of chloroform, methanol and 0.2% formic acid in water according to a modified procedure originally described by Bligh and Dyer [64]. The organic phase was collected, dried under nitrogen, and dissolved with 100 μl chloroform. The extract was spotted onto 0.25 mm silica gel HPTLC plates (Macherey-Nagel, Düren, Germany) and separated by thin-layer chromatography using the solvent system of ethyl acetate : iso-octane : H_2_O : acetic acid at 10:5:10:2 (v:v:v:v). The location of [^14^C]-labelled arachidonic acid and its metabolites was visualized and quantified using the phosphoimager system Cyclone^® ^Plus equipped with the software OptiQuant Acquisition and Analysis (Perkin Elmer, Rodgau-Jügesheim, Germany). To identify the spots of free arachidonic acid and its metabolites, non-radioactive standards were run in the same solvent system and visualized by exposing the plates to 10% phosphomolybdic acid in ethanol. Since PGE_2 _and TXB_2 _showed equal retention factor (Rf) values under these conditions, these metabolites were quantified as a single spot.

### Detection of 8-isoprostane

After treatment of cells the medium was collected, centrifuged for 5 min at 4°C and 13,000 × g to remove cell debris and stored at -80°C until analysis. 8-isoprostane was analyzed by a competitive enzyme immunoassay (EIA) according to the instructions of the manufacturer (Cayman, distributed by Biozol, Eching, Germany).

### Western blotting

Cells were harvested, washed twice with PBS and lysed for 20 min at 4°C in 75 μl RIPA lysis buffer (150 mM NaCl, 50 mM Tris-HCl pH 7.4, 5 mM EDTA, 1% (v/v) NP-40, 2 μg/ml leupeptin, 2 μg/ml aprotinine, 1 mM PMSF). For the detection of phosphorylated proteins, the lysis buffer additionally contained phosphatase inhibitor cocktail 1 and 2 (Sigma, Taufkirchen, Germany). The cell lysates were centrifuged at 12,000 g for 5 min at 4°C and the protein contents of the supernatants were measured with the BCA assay using BSA as a standard (Perbio, Bonn, Germany). Equivalent amounts of protein were loaded on a 10% sodium dodecyl sulphate (SDS)-polyacrylamide gel. After electrophoresis, the proteins were transferred to an Immobilon-FL or Immobilon-P PVDF membrane (Millipore, Eschborn, Germany). The membranes were blocked with 5% (w/v) non-fat dry milk in Tris-buffered saline (TBS) for 1 h and then incubated overnight at 4°C with the appropriate primary antibody in 5% non-fat dry milk in TBS containing 0.1% Tween20 (TBS-T). After washing, the membranes were either incubated with a secondary antibody coupled with horseradish peroxidase for detection with ECL reagents or to IRDye800 or Alexa680 coupled secondary antibodies for detection with the Odyssey Infrared Imaging System (LICOR Biosciences, Bad Homburg, Germany). The procedures for detection with ECL and the Odyssey method were performed in accordance with the manufacturer's instructions.

### Statistical analysis

Values are reported as mean ± standard error of the mean (s.e.m.) of several independent experiments as indicated in the legends. Statistical analysis was performed using Student's *t *test. Values of p < 0.05 were considered statistically significant.

## Competing interests

The authors declare that they have no competing interests.

## Authors' contributions

SFD, CW and SD conceived and designed the experiments. SFD and TB performed the experiments and analyzed the data. SM provided the MAF02 fly ash and analyzed the particle size distribution and both, SM and HRP contributed to discuss the particle data. SFD wrote the manuscript with assistance of CW and SD. All authors read and approved the final manuscript.
